# Air pollutants, seasonal influenza, and acute otitis media in children: a population-based analysis using 22-year hospitalization data

**DOI:** 10.1186/s12889-024-18962-4

**Published:** 2024-06-13

**Authors:** Conglu Li, Xiaoting Jiang, Yuchen Wei, Yawen Wang, Xiangqian Lao, Qianying Yue, Ka Chun Chong

**Affiliations:** 1 Jockey Club School of Public Health and Primary Care, The Chinese University of Hong Kong, Hong Kong Special Administrative Region, China; 2 Clinical Trials and Biostatistics Laboratory, Shenzhen Research Institute, The Chinese University of Hong Kong, Shenzhen, China; 3 Centre for Health Systems and Policy Research, The Chinese University of Hong Kong, Hong Kong Special Administrative Region, China; 4 Department of Biomedical Sciences, City University of Hong Kong, Hong Kong Special Administrative Region, China

**Keywords:** Influenza, Acute otitis media, Air pollutants, Meteorological factors, Children

## Abstract

**Background:**

Acute otitis media (AOM) is a prevalent childhood acute illness, with 13.6 million pediatric office visits annually, often stemming from upper respiratory tract infections (URI) and affected by environmental factors like air pollution and cold seasons.

**Methods:**

Herein, we made use of territory-wide hospitalization data to investigate the relationships between meteorological factors, air pollutants, influenza infection, and AOM for children observed from 1998 to 2019 in Hong Kong. Quasi-Poisson generalized additive model, combined with a distributed-lag non-linear model, was employed to examine the relationship between weekly AOM admissions in children and weekly influenza-like illness-positive (ILI +) rates, as well as air pollutants (i.e., oxidant gases, sulfur dioxide, and fine particulate matter), while accounting for meteorological variations.

**Results:**

There were 21,224 hospital admissions due to AOM for children aged ≤ 15 years throughout a 22-year period. The cumulative adjusted relative risks (ARR) of AOM were 1.15 (95% CI, 1.04–1.28) and 1.07 (95% CI, 0.97–1.18) at the 95th percentile concentration of oxidant gases (65.9 ppm) and fine particulate matter (62.2 μg/m^3^) respectively, with reference set to their medians of concentration. The ARRs exhibited a monotone increasing trend for all-type and type-specific ILI + rates. Setting the reference to zero, the cumulative ARRs of AOM rose to 1.42 (95% CI, 1.29–1.56) at the 95th percentile of ILI + Total rate, and to 1.07 (95% CI, 1.01–1.14), 1.19 (95% CI, 1.11–1.27), and 1.22 (95% CI, 1.13–1.32) for ILI + A/H1N1, A/H3N2, and B, respectively.

**Conclusions:**

Our findings suggested that policy on air pollution control and influenza vaccination for children need to be implemented, which might have significant implications for preventing AOM in children.

**Supplementary Information:**

The online version contains supplementary material available at 10.1186/s12889-024-18962-4.

## Background

Acute otitis media (AOM) is an infection that occurs in the middle ear usually accompanied by pain [1]. AOM is a common childhood disease with one in five children having at least an episode of AOM by the age of one. Approximately 60% of children will experience AOM at least one time by the age of three [2]. The high incidence rate of AOM imposes a significant burden on medical expenditure. In the United States, AOM accounts for an annual outpatient cost of $314 per child, and is associated with an extra medical expenditure of $2.88 billion per year [3]. Moreover, as most AOM cases are associated with bacterial infection such as *Streptococcus pneumoniae*, *Haemophilus influenzae*, and *Moraxella catarrhalis*, [4] more than 70% AOM children are prescribed with antibiotics [5, 6], raising serious concern to antimicrobial resistance [7, 8]. The occurrence of AOM presents an extensive seasonal variation, with 66% of visits identified between November and April of each year [9], parallel to the influenza seasons.

Influenza infection is a risk factor for AOM occurrence. Most cases of AOM are preceded by a viral upper respiratory tract infection (URI). Such infection could subsequently induce inflammation of the Eustachian tube and negative middle ear pressure. These conditions thus facilitate the entrance of secretions containing both colonized infecting virus and pathogenic bacteria from the nasopharynx into the middle ear [10]. Common types of viruses associated with AOM include respiratory syncytial virus, adenovirus, rhinovirus, and influenza virus [11]. According to Nokso-Koivisto *et al.*, of 303 URI episodes associated with single virus, the rate of AOM complicating URI associated with influenza virus was 31% [12]. Annual influenza vaccination is recommended for children older than six months [13] as it results in a 4% absolute reduction in AOM episodes and a 30% to 55% reduction in AOM during the respiratory illness season [14, 15].

The increased AOM risk under exposure to high concentration of air pollutants was supported by several epidemiological studies. Zemek R et al. studied the relationship between the incidence of AOM and air pollutants in children population, and suggested an exposure to nitrogen dioxide (NO_2_) was associated with the incidence of AOM [16]. Wang et al. revealed that AOM outpatient visit was positively correlated with sulfur dioxide (SO_2_), while ozone (O_3_) exhibited a negative correlation with it [17]. Apart from that, a retrospective study also found that particulate matters (i.e., PM_2.5_ and PM_10_) have adverse effects on AOM in children [18].

Although the relationship between air pollution and AOM in children was well supported by a wealth of evidence, to our knowledge, there are limited studies examining the effects of multiple exposome, including both pollutants and seasonal influenza on AOM at the population level. With the comprehensive population-based hospitalization records spanning from 1998 to 2019, our study aims to elucidate the relationships between air pollutants, meteorological factors, seasonal influenza, and AOM in children in Hong Kong, a non-industrial influenza epicenter.

## Methods

### Data sources

#### Hospital admission data

We collected weekly hospital admission data for children aged 0 to 15 years who were diagnosed with AOM from all public hospitals in Hong Kong, using the aggregated data provided by the Hong Kong Hospital Authority. The investigation data were obtained for the period between January 1, 1998, and December 31, 2019. Diagnoses of AOM for hospital admissions were identified based on codes 381–382 from the International Classification of Diseases, Ninth Revision, Clinical Modification (ICD-9-CM).

#### Air pollutants data

We collected weekly average levels of air pollutants from the Environment Protection Department, including NO_2_, SO_2_, O_3_, and PM_2.5_, which were measured by 13 general air monitoring stations namely Central/Western, Eastern, Kwai Chung, Kwun Tong, Sham Shui Po, Sha Tin, Tai Po, Tap Mun, Tseng Kwan O, Tsuen Wan, Tuen Mun, Tung Chung, and Yuen Long. However, Tap Mun station, located in a remote area with low population density, was excluded from the analysis. The pollutant concentrations were then averaged on a weekly basis using data from the remaining 12 general air monitoring stations.

We used redox-weighted oxidant capacity (O_x_), calculated as O_x_ = (1/3) NO_2_ + (2/3) O_3_, to represent the combined oxidative effect of NO_2_ and O_3_ [19]_,_ which has been used in similar studies [20, 21]. The redox-weighted oxidant capacity was proven to be an alternative proxy for atmospheric oxidation capacity, which is better than the unweighted oxidant capacity [22].

#### Meteorological data

The data of weekly mean ambient temperature, mean relative humidity, and total rainfall were obtained from the Hong Kong Observatory, and they were measured in the station of the Hong Kong Observatory Headquarters.

#### Influenza data

Weekly consultation rates of influenza-like illness (ILI) were obtained from a local sentinel surveillance system which contains 64 general outpatient clinics and over 40 private medical practitioner clinics from the Center for Health Protection, Hong Kong. The surveillance system reported the proportion of outpatient consultations with cough, fever > 38.5℃, and sore throat on a weekly basis. Surveillance data on influenza virus isolation were obtained from the Public Health Laboratory Service of the Centre for Health Protection. Outpatient clinics and inpatient hospitals regularly submitted respiratory specimens to the Branch for surveillance and diagnosis. The number of test specimens was collected, and the number of influenza-positive specimens was classified by type and subtype. We further determined the influenza-like illness-positive (ILI +) rate as a proxy for influenza activity by multiplying the proportion of ILI consultations by the proportion of positive respiratory specimens for a specific influenza subtype. The proportion of ILI consultations reflects the changing numbers of sick individuals sampled for testing, while the viral positivity indicator accounts for variation in the proportion of ILI cases that are attributable to influenza infection. Consequently, the use of ILI + rates can not only reduce the sampling uncertainty of laboratory testing when there are fewer samples to be tested during periods of non-influenza epidemics, but also reduce the number of over-diagnoses from clinical consultations [23–26]. The aggregation time series of ILI + A/H1N1, A/H3N2, and B, as well as the combined rate including all influenza strains (i.e., ILI + Total) were obtained.

### Statistical methods

To examine the effects of air pollutants, weather factors, and influenza on AOM, we used quasi-Poisson generalized additive models in combination with the distributed lag non-linear model (DLNM) [27–29]. The full form of the model is as follows:

$$\text{log}\left(\mu_t\right)=intercept+cb\left(pollutant_t;lag\right)+cb\left(temp_t;\left.lag\right)\right.+cb\left(humid_t\right.;\left.lag\right)+cb\left(rain_t\right.;\left.lag\right)+cb\left(ILI+Total_t\right.;\left.lag\right)+s\left(year_t\right)+s\left(week_t\right)+offset_t+Autoregressive terms$$

where *μ*_*t*_ is the expected number of AOM admissions in a given week. The cross-basis function *cb*(.) is employed to model both the relationship between exposure and response, as well as delayed effects. Function *s*(.) represents a smoothing spline function. As PM_2.5_, SO_2_, and O_x_ are highly correlated with each other, the effect of these air pollutants was examined individually as the term *pollutant*_*t*_ in the model to avoid collinearity. The mean temperature (*temp*_*t*_), relative humidity (*humid*_*t*_), total rainfall (*rain*_*t*_), and the rate of ILI + Total (*ILI* + *Total*_*t*_) at week *t* were included in the model. *Year*_*t*_ and *week*_*t*_ denote the year and the week of the year, respectively, which were used to capture long-term trends and seasonal patterns. The offset term (*offset*_*t*_) represents the natural logarithm of the total number of hospital admissions for all causes in week *t*. The degrees of freedom of the exposure variables and lag parameters in the *cb*(.) functions were determined through the minimization of the generalized cross-validation statistic, with a maximum lag of 2 weeks. The assumption of maximum lag was additionally tested by altering it to 1 week and 3 weeks.

We examined the association of each influenza type by substituting the term *ILI* + *Total*_*t*_ with a combination of the terms *ILI* + *A/H1N1*_*t*_*, ILI* + *A/H3N2*_*t*_*, and ILI* + *B*_*t*_*,*

$$\text{log}\left({\mu }_{t}\right)=intercept+cb\left(pollutant_{t}\right.;\left.lag\right)+cb\left(temp_{t}\right.;\left.lag\right)+cb\left(humid_{t}\right.;\left.lag\right)+cb\left(rain_{t}\right.;\left.lag\right)+cb\left(ILI+ A/H1N1_{t}\right.;\left.lag\right)+cb\left(ILI+ A/H3N2_{t}\right.;\left.lag\right)+cb\left(ILI+ B_{t}\right.;\left.lag\right)+s\left(year_{t}\right)+s\left(week_{t}\right)+offset_{t}+Autoregressive terms$$

The exposure effects were quantified using cumulative adjusted relative risk (ARR) over the lag period, accompanied by the corresponding 95% confidence intervals (CIs). The medians of air pollutants and meteorological variables such as mean temperature and relative humidity served as their respective reference values, while zero was set as the reference value for ILI + rates and rainfall.

In light of the previous outbreaks of severe acute respiratory syndrome (SARS) in 2003 and swine-originated influenza A (H1N1) in 2009 experienced in Hong Kong, we conducted a sensitivity analysis by excluding data from these two years to account for any potential disruptions in hospitalization patterns and ensure the robustness of the results.

All the statistical analyses were performed in the R environment (version 4.0.3; ) using the “dlnm” and “mgcv” packages.

## Results

Descriptive statistics of the weekly hospital admissions due to AOM, ILI + rates, meteorological variables, and air pollutants from 1998 to 2019 in Hong Kong are presented in Table 1, and the temporal trends of ILI + rates and environmental factors are shown in Figure S1. There were 21,224 hospital admissions for AOM throughout the study period, with a median (IQR) of 16 (12–23) AOM admissions per week. The median rate (IQR) of ILI + Total was 3.40 (1.23–8.02) per 1,000 consultations, with ILI + A/H3N2 being the dominant strain. Pearson correlation coefficients between air pollutants are presented in Table S1.

**Table 1:** Descriptive statistics of the weekly total number of hospital admissions due to acute otitis media, ILI + rates, meteorological variables, and air pollutants over 1998–2019 in Hong Kong

		5th percentile	25th percentile	Median	75th percentile	95th percentile
Weekly AOM admission number	All	7	12	16	23	38
Weekly ILI + rates (per 1,000 consultations)	ILI + Total	0.37	1.23	3.40	8.02	19.91
	ILI + A/H1N1	0.00	0.00	0.22	1.06	7.06
	ILI + A/H3N2	0.00	0.28	0.97	3.68	14.00
	ILI + B	0.00	0.15	0.45	1.38	5.08
Environmental covariates	Mean temperature (°C)	15.08	19.49	24.54	27.79	29.69
	Mean relative humidity (%)	62.71	74.36	79.57	83.57	88.67
	Total rainfall (mm)	0.00	0.30	11.40	57.00	200.87
	O_x_ (μg/m^3^)	22.83	32.79	42.81	53.04	65.91
	SO_2_ (μg/m^3^)	5.62	9.15	13.15	18.58	27.82
	PM_2.5_ (μg/m^3^)	10.94	20.57	30.34	42.74	62.18

*AOM* Acute otitis media, *ILI* + Influenza-like illness-positive, *O*_*x*_ redox-weighted oxidant capacity, *SO*_*2*_ Sulfur dioxide, *PM*_*2.5*_ fine particulate matter

**Fig. 1:**
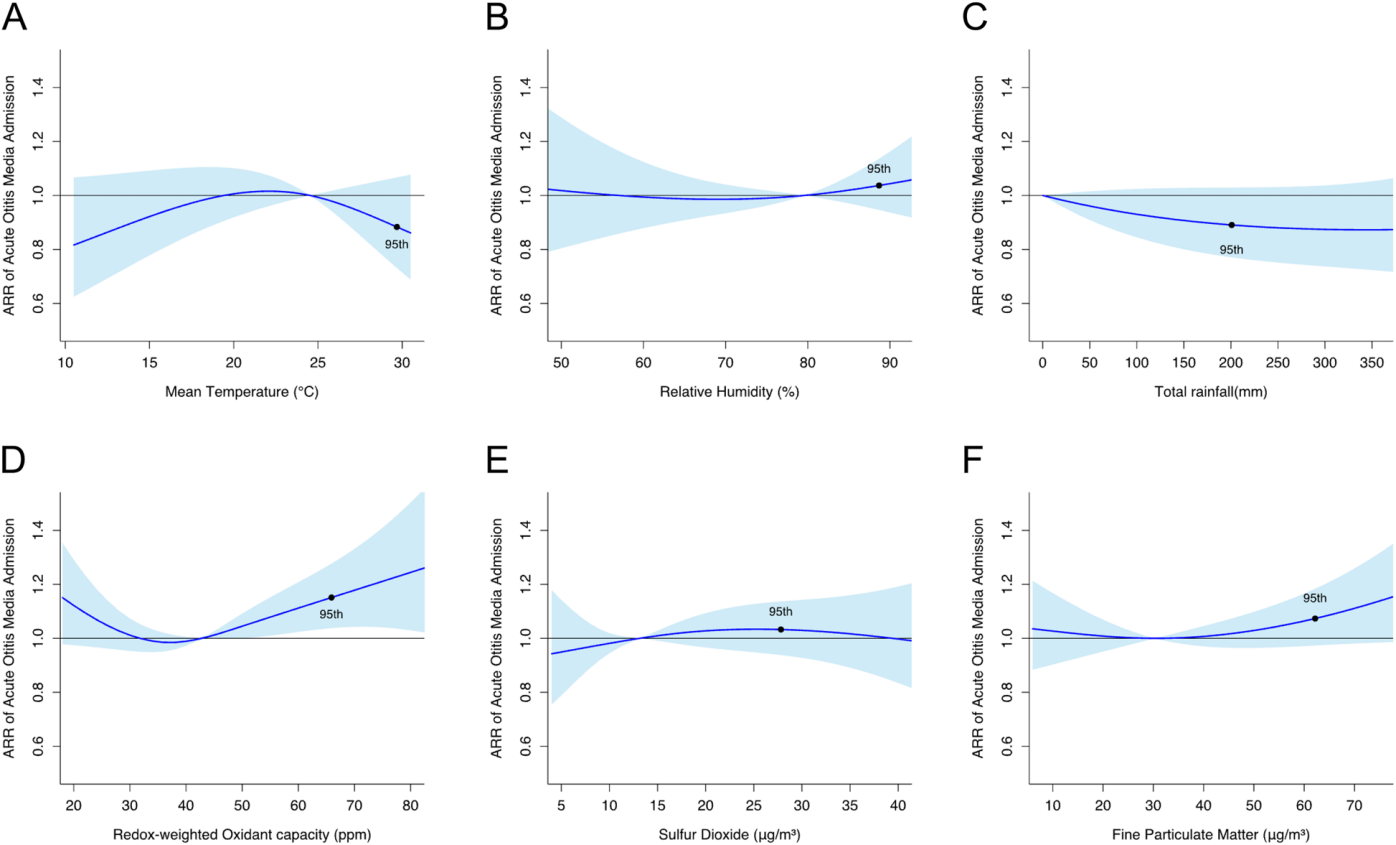
Cumulative adjustive relative risks (ARRs) with 95% confidence intervals on acute otitis media admissions against different environmental variables. The cumulative ARRs at the 95th percentiles of the environmental variables were dotted in the figures. The reference values were set as zero for total rainfall, and medians for other environmental variables

A high concentration of O_x_, among air pollutants examined, exhibited a statistically significant association with an increased risk of AOM admissions (Fig. 1). The cumulative ARR of admission at the 95th percentile of O_x_ concentration (65.9 ppm) was 1.15 (95% CI, 1.04–1.28), using the median O_x_ concentration (42.8 ppm) as the reference value. Similarly, the 95th percentile of PM_2.5_ concentration (62.2μg/m^3^) was marginally significantly associated with AOM, with a cumulative ARR of 1.07 (95% CI, 0.97–1.18) compared to the median level (30.3μg/m^3^). However, no significant associations were found between AOM and SO_2_ or meteorological factors such as ambient temperature, relative humidity, and total rainfall.

**Fig. 2:**
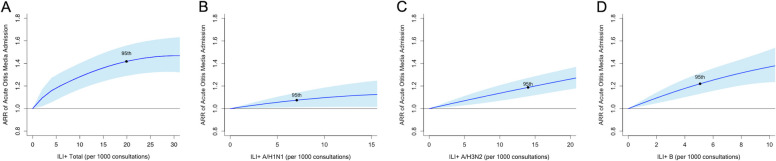
Cumulative adjustive relative risks (ARRs) with 95% confidence intervals on acute otitis media admissions against different influenza-like illness (ILI) + rates. The cumulative ARRs at the 95th percentiles of ILI + rates were dotted in the figures. The reference values were set as zero

The rates of ILI + Total, ILI + A/H1N1, ILI + A/H3N2, and ILI + B were all found to be significantly associated with an increased risk of AOM admissions (Fig. 2). When the reference rate for ILI + Total was set to zero, the cumulative ARR of AOM admissions rose to 1.42 (95% CI, 1.29–1.56) at the 95th percentile of ILI + Total rate. In addition, the cumulative ARRs increased to 1.07 (95% CI, 1.01–1.14), 1.19 (95% CI, 1.11–1.27), and 1.22 (95%CI, 1.13–1.32) for ILI + A/H1N1, A/H3N2, and B, respectively, when their rates reached the 95th percentile.

In the sensitivity analyses, although excluding data from 2003 and 2009 led to slight alterations in the cumulative ARRs of the variables of interest, their significance remained unchanged (Figure S2 and S3).

## Discussion

In this study, we elucidate the relationships between multiple environmental factors, seasonal influenza, and AOM in children in Hong Kong. According to our results, a high concentration of PM_2.5_ and oxidant gases increased the risk of AOM hospitalization in pediatric patients. Previous studies have reported air pollutants influence the occurrence of otitis media (OM), but the results were generally inconsistent [16, 30–32]. A study conducted in Canada suggested that exposure to carbon monoxide and NO_2_ were positively related to AOM, especially in warmer months, while PM_2.5_ was not found to be a risk factor [16]. Nevertheless, some investigations reported a strong link between OM and multiple air pollutants, particularly in the warm seasons for children with a recent history of URI [31]. We speculate that the variation in levels of air pollutants, individual exposure patterns, and the socioeconomic characteristics of the participants may contribute to the inconsistent results among the studies [33].

We showed that oxidant gases were positively associated with the risk of AOM hospitalization in children. In fact, many in vivo and in vitro studies suggested plausible pathophysiological mechanisms for the relationship. Firstly, short-term exposure to NO_2_ results in a significant reduction in mucociliary activity, which may disrupt mucociliary clearance in the middle ear and upper airway. This ultimately leads to eustachian tube dysfunction, which causes middle ear fluid stasis and subsequent middle ear infections [34, 35]. Secondly, acute exposure to oxidant gases induces ciliostasis in the upper and lower airways, preventing the nasal and bronchial mucosa from filtering inhaled particles, such as airborne allergens, bacteria, or viruses, which makes children more susceptible to viral diseases of the upper respiratory tract, thus resulting in AOM [36, 37]. Meanwhile, oxidant gases can amplify the production of inflammatory cytokines by infected cells, exacerbating virus-induced inflammation of the respiratory system [38–40].

In our study, an increased PM_2.5_ was marginally associated with a higher risk of AOM in the children population. Literature has indeed suggested potential pathophysiological mechanisms to explain the association. Inhalation of PM_2.5_ may lead to particulate matters passing through the nasopharyngeal cavity and entering the airways and lungs, where the expression of genes associated with the inflammatory response is upregulated and mucus protein secretion is stimulated [41, 42]. Increased mucus proteins and inflammatory responses are capable of narrowing or blocking the eustachian tube, allowing pathogen growth and susceptibility to middle ear inflammation. Moreover, exposure to PM_2.5_ may further reduce mucus clearance by cilia, leading to inflammation and increased susceptibility to AOM [43].

In this study, we showed that seasonal influenza was associated with an increased risk of AOM admissions. Not surprisingly, influenza and other respiratory viruses are known to be common causes of AOM. Upper respiratory tract infection causes congestion of the nasopharyngeal mucosa and inflammation of the eustachian tube [44]. This can lead to eustachian tube dysfunction, which is thought to be the most important factor in the development of AOM [45]. Apart from that, studies have demonstrated that respiratory viruses promote the release of inflammatory mediators, some of which (e.g., histamine, kinins) can induce eustachian tube dysfunction [46]. Eustachian tube dysfunction hinders the clearance of middle ear secretions, leading to fluid accumulation. Bacteria and viruses can easily grow in the accumulated fluid, thus causing acute otitis media. In spite of a strong link between influenza and AOM, the risk of influenza A/H1N1 was lower than that of A/H3N2 and B. We speculate that the variation of susceptibility by influenza type may influence the magnitude of risk for AOM incidents [47].

While studies demonstrated that meteorological factors could be related to the incidence of AOM [48], we did not identify any significant meteorological factors as risk factors. In fact, dry and cold conditions were suggested to cause dryness of the nasal and nasopharyngeal mucosa and reduced surface ciliary activity, increasing the spread of pathogens [48]. As the incidence of influenza also peaks in cold seasons, we suspect that the elevated risk of AOM in cold temperatures may be due to the modulating effect of influenza infection.

This study also has some limitations. Firstly, it is a modelling study using aggregate data to draw relevant conclusions, so ecological fallacies may occur. Secondly, in our data analysis, we didn’t take the influenza vaccination rate into account, while the vaccination uptake rate was low during the study period [49].

## Conclusions

We demonstrated that exposure to PM_2.5_, oxidant gases, and seasonal influenza were risk factors for AOM in children in Hong Kong, a non-industrial influenza epicenter. Given the high incidence of AOM in children and the ubiquitous characteristic of air pollutant exposure, influenza vaccination and policy on air pollution control are necessary to be implemented.

### Supplementary Information

Additional file 1: Figure S1. Temporal trends of influenza-like illness-positive (ILI+) rates and environmental variables in Hong Kong from 1998 to 2019.

Additional file 2: Figure S2. Sensitivity analysis for impacts of environmental variables when data from 2003 and 2009 were removed. The cumulative adjustive relative risks (ARRs) at the 95th percentiles of the environmental variables were dotted in the figures. The reference values were set as zero for total rainfall, and medians for other environmental variables.

Additional file 3: Figure S3. Sensitivity analysis for impacts of different influenza-like illness-positive (ILI+) rates when data from 2003 and 2009 were removed. The cumulative adjustive relative risks (ARRs) at the 95th percentiles of ILI+ rates were dotted in the figures. The reference values were set as zero.

Additional file 4: Table S1. Pearson correlation coefficient between different air pollutants during the study period (1998-2019).

## Data Availability

The data that support the findings of this study are available from the Hospital Authority, The Government of the Hong Kong Special Administrative Region but restrictions apply to the availability of these data, which were used under license for the current study, and so are not publicly available. Data are however available from the authors upon reasonable request and with permission of the Hospital Authority.

## Declarations

** Ethics approval and consent to participate**

This was an observational study using aggregate data provided and permitted to use by the Hospital Authority, The Government of the Hong Kong Special Administrative Region. The data used in this study was anonymized before its use. Either ethics approval or informed consent was waived.

** Consent for publication**

NA.
