# Supplementary figures and images for: Air pollutants, seasonal influenza, and acute otitis media in children: a population-based analysis using 22-year hospitalization data
Source: BMC Public Health. 2024 Jun 13;24:1581. doi: 10.1186/s12889-024-18962-4 (PMC11170825; doi:10.1186/s12889-024-18962-4)

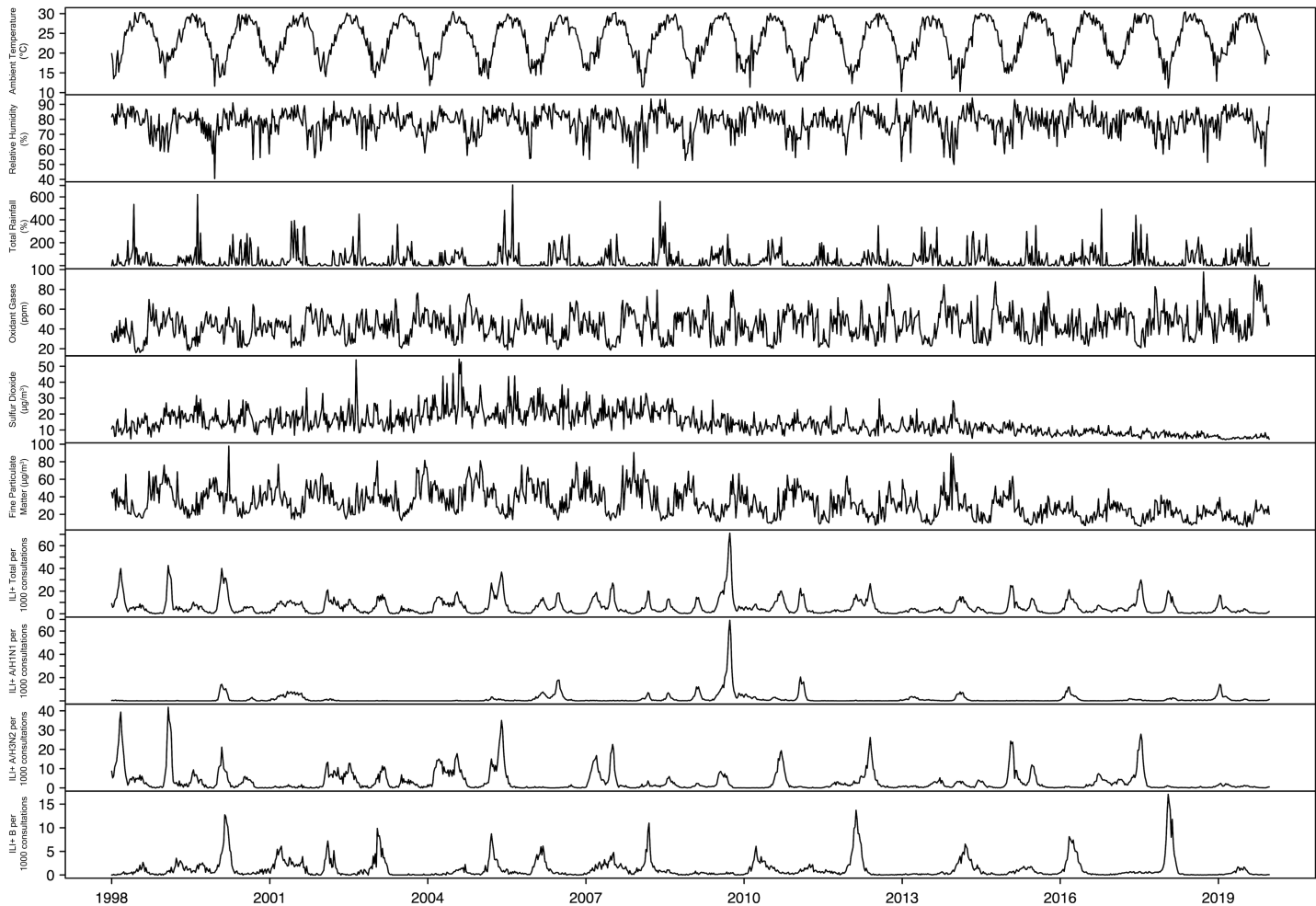

Supplement: Supplementary file 1 — Additional file 1: Figure S1. Temporal trends of influenza-like illness-positive (ILI+) rates and environmental variables in Hong Kong from 1998 to 2019. [file 12889_2024_18962_MOESM1_ESM.pdf]

A

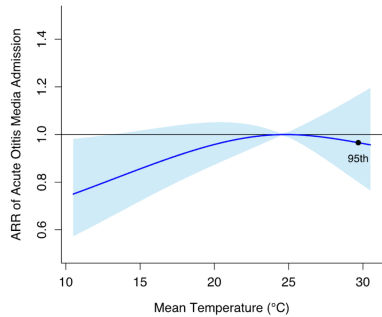

B

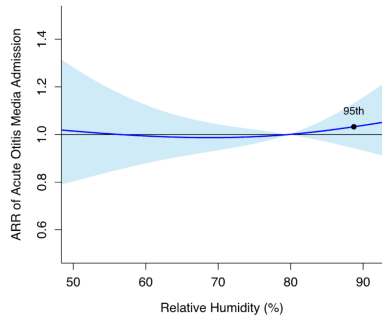

C

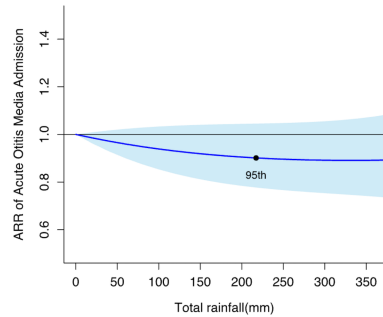

D

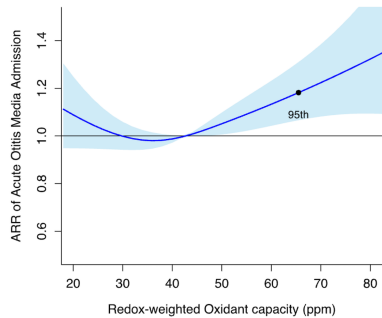

E

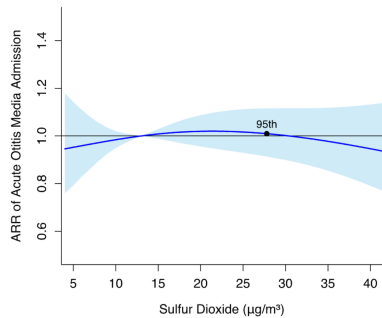

F

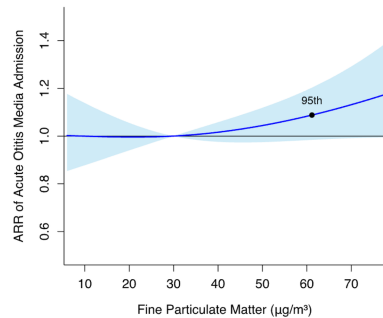

Supplement: Supplementary file 2 — Additional file 2: Figure S2. Sensitivity analysis for impacts of environmental variables when data from 2003 and 2009 were removed. The cumulative adjustive relative risks (ARRs) at the 95th percentiles of the environmental variables were dotted in the figures. The reference values were set as zero for total rainfall, and medians for other environmental variables. [file 12889_2024_18962_MOESM2_ESM.pdf]

A

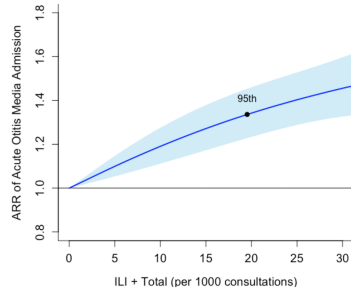

B

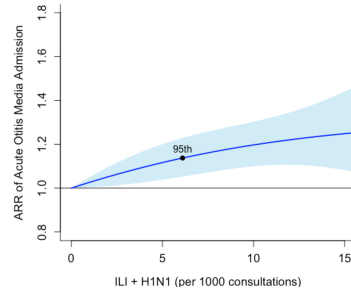

C

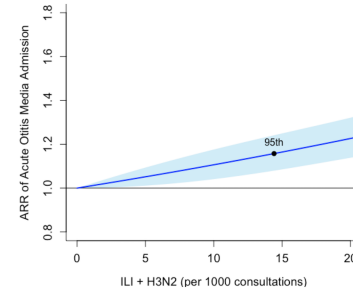

D

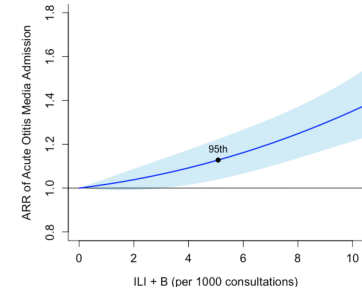

Supplement: Supplementary file 3 — Additional file 3: Figure S3. Sensitivity analysis for impacts of different influenza-like illness-positive (ILI+) rates when data from 2003 and 2009 were removed. The cumulative adjustive relative risks (ARRs) at the 95th percentiles of ILI+ rates were dotted in the figures. The reference values were set as zero. [file 12889_2024_18962_MOESM3_ESM.pdf]
