# Supplementary material for: Air pollutants, seasonal influenza, and acute otitis media in children: a population-based analysis using 22-year hospitalization data
Source: BMC Public Health. 2024 Jun 13;24:1581. doi: 10.1186/s12889-024-18962-4 (PMC11170825; doi:10.1186/s12889-024-18962-4)
Supplement: Supplementary file 4 — Additional file 4: Table S1. Pearson correlation coefficient between different air pollutants during the study period (1998-2019). [file 12889_2024_18962_MOESM4_ESM.docx]

**Table S1. Pearson correlation coefficient between different air pollutants during the study period (1998-2019).**

|  | NO_2_ | O_3_ | SO_2_ | PM_2.5_ |
| --- | --- | --- | --- | --- |
| NO_2_ |  | 0.296 | 0.440 | 0.817 |
| O_3_ |  |  | -0.183 | 0.370 |
| SO_2_ |  |  |  | 0.514 |
| PM_2.5_ |  |  |  |  |
